# Hollow-Fiber RO Membranes Fabricated via Adsorption of Low-Charge Poly(vinyl alcohol) Copolymers

**DOI:** 10.3390/membranes11120981

**Published:** 2021-12-15

**Authors:** Takashi Ohkame, Kazushi Minegishi, Hideki Sugihara, Keizo Nakagawa, Takuji Shintani, Hideto Matsuyama, Tomohisa Yoshioka

**Affiliations:** 1 Research Center, Toyobo Co., Ltd., 1-1 Katata 2-Chome, Otsu 520-0292, Japan; kazushi_minegishi@toyobo.jp (K.M.); hideki_sugihara@toyobo.jp (H.S.); 2 Graduate School of Science, Technology and Innovation, Kobe University, 1-1 Rokkodai, Nada, Kobe 657-8501, Japan; k.nakagawa@port.kobe-u.ac.jp (K.N.); shintani@port.kobe-u.ac.jp (T.S.); 3 Research Center for Membrane and Film Technology, Kobe University, 1-1 Rokkodai, Nada, Kobe 657-8501, Japan; matuyama@kobe-u.ac.jp

**Keywords:** reverse osmosis, hollow-fiber membrane, layer-by-layer, poly(vinyl alcohol) copolymer

## Abstract

We report a new type of alkaline-stable hollow-fiber reverse osmosis (RO) membrane with an outside-in configuration that was established via adsorption of positively charged poly(vinyl alcohol) copolymers containing a small amount of quaternary ammonium moieties. Anionic sulfonated poly(arylene ether sulfone nitrile) hollow-fiber membranes were utilized as a substrate upon which the cationic copolymer layer was self-organized via electrostatic interaction. While the adsorption of the low-charge copolymer on the membrane support proceeded in a Layer-by-Layer (LbL) fashion, it was found that the adsorbed amount by one immersion step was enough to form a defect-free separation layer with a thickness of around 20 nm after cross-linking of vinyl alcohol units with glutaraldehyde. The resultant hollow-fiber membrane showed excellent desalination performances (NaCl rejection of 98.3% at 5 bar and 1500 mg/L), which is comparable with commercial low-pressure polyamide RO membranes, as well as good alkaline resistance. The separation performance could be restored by repeating the LbL treatment after alkaline degradation. Such features of LbL membranes may contribute to extending RO membrane lifetimes.

## 1. Introduction

Reverse osmosis (RO) is now a widely accepted desalination process. According to the forecast by Global Water Intelligence’s DesalData, the worldwide operating RO plant capacity reached 58.5 Mm^3^/day in 2020 and is expected to reach 82.1 Mm^3^/day in 2026. Although RO has emerged as one of the most important technologies to tackle water scarcity throughout the world, more energy-efficient RO processes and mitigation of environmental risks caused by brine discharges are required to achieve sustainable desalination [1]. The development of long-life RO membranes with higher chemical stability is essential to reduce consumption of chemicals and minimize membrane replacement in RO processes. Currently, there are two types of commercially available RO membrane materials, namely cross-linked aromatic polyamide comprised of *m*-phenylenediamine, trimesoyl chloride and cellulose triacetate. These two materials have been far superior in desalination performance and chemical stability compared with other synthetic polymer materials that have been investigated in the last half century [2]. However, polyamide (PA) is susceptible to low levels of free chlorine, which leads to frequent membrane cleanings and shortening of membrane life because of severe biofouling [3]. It is known that chlorination of the amide bond (*N*-chlorination) promotes alkaline hydrolysis of PA, which causes irreversible membrane degradation [4,5]. In contrast, cellulose triacetate (CTA) is a chlorine-tolerant material and chlorine injection during RO operation enables significant suppression of biofouling [6]. However, it is known that CTA undergoes hydrolysis in an alkaline medium. Once these membranes suffer degradation, the membrane performances cannot be restored, and membrane replacement becomes necessary.

In this regard, layer-by-layer (LbL) deposition is a promising method that has attracted much attention for fabrication of self-assembled nanolayers for use in various membrane separation applications, such as nanofiltration, forward osmosis, and gas separation [7,8,9,10,11,12,13]. In the LbL process, oppositely charged polyelectrolytes are alternately deposited onto a charged membrane substrate [14]. Given that the LbL layer is formed via an ion exchange mechanism between counter ions of charged substrates and fixed ions of polyelectrolytes [15], the formation of LbL separation layer on charged substrates is considered to be essentially reversible and regenerable as long as the surface charges of substrates are not lost [16]. Such regenerability of LbL membranes may contribute to extending membrane life. However, one drawback of LbL membranes is their low physical durability because of the intrinsic water solubility of highly charged polyelectrolytes. Cross-linking of the polyelectrolyte adsorption layer significantly improves the durability and separation performance of LbL membranes [10,11]. Duong et al. [10] fabricated an adsorption layer comprised of poly(allylamine hydrochloride) (PAH) and poly(sodium 4-styrene sulfonate) (PSS) on a hydrolyzed poly(acrylonitrile) (PAN) membrane and cross-linked PAH layers with glutaraldehyde and PSS layers by UV exposure. Liu et al. [11] fabricated a poly(ethersulfone) (PES) hollow-fiber membrane and coated PAH and PSS on the inner surface of the membrane followed by cross-linking of PAH layer with glutaraldehyde. In their works, ionic strength and pH of polyelectrolyte aqueous solutions were carefully controlled and high NaCl concentration (typically 0.5–1.0 M) was maintained. The salt addition is important to screen the strong intramolecular and intermolecular electrostatic forces of highly charged polyelectrolytes [10]. pH is also an important parameter for controlling the degree of dissociation of fixed weak-charge groups (e.g., carboxylic or amino group) when weak polyelectrolytes are adopted. The thickness of adsorption layers of high-charge polyelectrolytes increases with increased salt concentration [17] and makes stepwise growth by alternate depositions of oppositely charged polyelectrolytes [12]. The original concept of the LbL accumulation under controlled ionic strength and pH has a drawback in the practical manufacturing process because of the necessity of a large number of iterative adsorption steps. However, to the best of our knowledge, utilization of low-charge copolymers containing a very small amount of charged moiety (ca. 1 mol%) has not been investigated for fabrication of LbL membranes. The advantage of utilizing such low-charge copolymers is their strong adsorption character at very low ionic strength conditions in one adsorption step. It is known that the adsorbed amounts of low-charge polyelectrolytes decrease with increasing ionic strength. This feature is typical of a “screening reduced adsorption” regime as described theoretically by van de Steeg et al. [18] and supported by several experimental results [19,20,21].

In this study, we examined a poly(vinyl alcohol-*co*-diallyldimethylammonium chloride) (CPVA) as a low-charge cationic polyelectrolyte for fabricating a separation layer adsorbed onto the anionic outer surface of hollow-fiber RO membrane. As an alkaline stable hollow-fiber support, polyphenylene oxide (PPO) hollow-fiber membrane coated with sulfonated poly(arylene ether sulfone nitrile) (SPN-20) was utilized. The amount of cationic moiety incorporated in the CPVA copolymer was so small (1.36 mol%) that the intramolecular repulsion in the copolymer is innately small and the maximum amount of adsorption could be obtained without specific control of salt concentration and pH. Cross-linking of hydroxyl groups of the adsorbed CPVA copolymer by glutaraldehyde led to a stable and tight RO separation layer. The surface characteristics and low-pressure RO separation performances of the hollow-fiber membranes are noted herein.

## 2. Experimental

### 2.1. Materials

Anionic hollow-fiber membrane support was obtained from Toyobo Research Center (Otsu, Japan). The membrane had a separation layer comprised of sulfonated poly(arylene ether sulfone nitrile) containing benzonitrile moieties (SPN-20), which was coated on a polyphenylene oxide (PPO) hollow-fiber support. The detailed properties of the SPN-20/PPO thin-film composite hollow-fiber membrane were described in our previous study [22]. The powder form of SPN-20 was also supplied by Toyobo Research Center. PPO powder was purchased from Sigma Aldrich (181781, St. Louis, MO, USA). Vinyl acetate (Nakalai Tesque, Kyoto, Japan) and diallyldimethylammonium chloride (TCI, Tokyo, Japan) were used as monomers for the synthesis of cationic poly(vinyl alcohol) copolymers. 2,2′-Azobis(isobutyronitrile) (AIBN, Fujifilm-Wako, Osaka, Japan) was used as radical initiator. Other chemicals were reagent grade and were used as received.

### 2.2. Synthesis and Characterization of CPVA Copolymers

Poly(vinyl alcohol-*co*-diallyldimethylammonium chloride) (CPVA) was synthesized via a radical copolymerization method [23,24]. PVA structure was chosen as a major component of the copolymer because PVA is a cost-effective and chemically stable polymer that can be easily cross-linked to form a stable three-dimensional network structure using aldehydes [25]. In addition, PVA is an intrinsically water-soluble polymer that is suitable for the LbL-type adsorption process in the aqueous phase. A small amount of cationic monomer, diallyldimethylammonium chloride (DADMAC), was copolymerized in PVA to provide copolymers with functionality for electrostatic adsorption. DADMAC was chosen because it forms a chemically stable, strongly basic five-membered pyrrolidinium ring and does not hydrolyze in the saponification step where vinyl acetate (VAC) is converted to vinyl alcohol (VA). Briefly, appropriate amounts of VAC, DADMAC, methanol, and AIBN were introduced into a 2-L reactor equipped with a condenser, an inlet of nitrogen and monomer solution, and a stirrer. The reaction was started at 60 °C under nitrogen atmosphere, and was carried out for 120 min. After completion of the reaction, the remaining monomer was distilled out and a sodium hydroxide solution in methanol (0.05 mol per vinyl acetate unit) was added and maintained at 40 °C under vigorous stirring to obtain completely hydrolyzed CPVA (degree of saponification > 99%). After washing polymers with methanol and drying at 60 °C in vacuum, the powder form of CPVA was obtained. The chemical structure of CPVA was determined by ^1^H nuclear magnetic resonance (NMR) spectroscopy (Varian 400 MHz, Agilent, Santa Clara, CA, USA) using D_2_O solvent. The weight average and the number average molecular weights (*M*_w_ and *M*_n_) were measured by gel permeation chromatography (GPC; HLC-8320GPC; columns: super-HM-H*2 + super H2000, Tosoh, Tokyo, Japan) based on polymethyl methacrylate standards. Hexafluoroisopropanol with 10 mM trifluoroacetic acid was used as solvent for GPC separations.

### 2.3. Adsorption Measurements of CPVA on Hollow-Fiber Supports

The adsorbed amounts of CPVA on hollow-fiber membrane supports were evaluated by measuring the change in concentration of CPVA aqueous solution before and after immersing hollow-fiber supports in copolymer solutions. A 20-m long hollow-fiber sample was cut into pieces and dispersed in 10 mL of CPVA aqueous solution in a vial at room temperature for 20 min. The initial CPVA concentration was set at 100 mg/L and ultra-pure water was used as solvent. Ionic strengths of the CPVA solutions were adjusted using NaCl. The concentrations of CPVA were measured by total organic carbon analysis (TOC; ON-LINE TOC-VCSH, Shimadzu, Kyoto, Japan) using a non-purgeable organic carbon (NPOC) method.

### 2.4. Fabrication of Cross-Linked CPVA (XCPVA)-Modified Hollow-Fiber Membranes

The LbL protocol of the CPVA adsorption and cross-linking by glutaraldehyde was determined by preliminary tests. The CPVA concentration *C*_p_ was optimized at *C*_p_ = 1000 mg/L. Briefly, when *C*_p_ was lower than 50 mg/L, the adsorbed amount of CPVA was not enough to cover all of the SPN-20 surface. When *C*_p_ was higher than 5000 mg/L, the permeate flux of the XCPVA membrane decreased, possibly because of the increased viscosity of CPVA solutions. CPVA (degree of cationization: 1.36 mol%) was dissolved in deionized water to prepare CPVA solution (1000 mg/L). A bundle of SPN-20/PPO hollow-fiber membranes (typically 400 fibers) in a plastic pipe module was filled with the CPVA solution for 30 min to form a CPVA adsorption layer on the SPN-20 surface in a layer-by-layer fashion. After the adsorption step, the sample was rinsed with deionized water for 1 min to remove excess polymer. Subsequently, the sample was filled with 1 wt% aqueous glutaraldehyde solution (pH 2, adjusted with HCl solution) for 24 h at room temperature to cross-link and insolubilize the CPVA adsorption layer. The sample was then thoroughly washed with deionized water and stored until use.

### 2.5. SEM and TEM Observation of Membrane Structures

The cross-sections of hollow-fiber membranes were observed by scanning electron microscopy (SEM, S-4800, Hitachi, Tokyo, Japan). SEM samples were obtained by cryogenic breaking of hollow-fiber samples in liquid nitrogen and the cross-section was sputtered with platinum before analysis. The CPVA separation layer formed on SPN-20/PPO hollow-fiber supports was partially cross-linked using titanium lactate (TC310, Matsumoto Fine Chemical, Ichikawa, Japan) to enhance the electron density contrast by incorporating heavy Ti elements into the CPVA layer. A hollow-fiber sample for observation by transmission electron microscopy (TEM) was embedded in a light-cured epoxy resin and microtomed in cross-section with a diamond knife and sputtered with carbon before analysis.

### 2.6. Membrane Surface Roughness Analyzed by AFM

The surface morphologies of the SPN-20/PPO hollow-fiber membrane and the XCPVA-modified hollow-fiber membrane were measured in the dynamic force mode (tapping mode) by atomic force microscopy (AFM; E-sweep/SPI4000 system, SII NanoTechnology, Tokyo, Japan) equipped with a 20-µm scanner and Si-DF3 cantilevers. AFM measurements were performed on hydrated surfaces of hollow-fiber membranes in aqueous medium. The average surface roughness (*R*_a_) calculated for a scan area of 2.0 µm ×  2.0 µm was utilized to compare the surface roughness of the samples.

### 2.7. Zeta Potentials of Polymeric Film Surfaces

The surface charge characteristics of the polymeric flat films of PPO, SPN-20, and XCPVA layer on SPN-20 film were investigated. To obtain polymeric films of PPO and SPN-20, 15 wt% of each polymer solution (PPO in chloroform and SPN-20 in *N*-methyl-2-pyrrolidone) was cast on a preheated glass plate using a film applicator and left to dry for 4 h. After peeling off, the film was completely dried in a vacuum oven at 120 °C. The XCPVA layer was prepared as follows. An air side of clean SPN-20 film was immersed in CPVA aqueous solution (1000 mg/L) for 30 min. After adsorption of CPVA, the film was immersed in deionized water and rinsed for 1 min, then immersed in 1 wt% glutaraldehyde solution (pH 2, adjusted with HCl solution) for 24 h at room temperature. The sample was washed with deionized water and left to dry under clean nitrogen flow. Zeta potentials of film surfaces were measured using an electrokinetic analyzer (SurPASS 3, Anton Paar, Graz, Austria). The measurements were performed using an adjustable gap cell with a rectangular size of 20 × 10 mm. Two clean flat-film samples were bonded on the top and bottom cell surfaces using double-sided tapes and mounted on the measurement system. The gap height was adjusted to 100 ± 5 μm. Zeta potential was measured as a function of pH in the range of pH 9 to 3 using 0.1 M KOH and 0.1 M HCl solutions. All measurements were performed using standard solution of 1 mM KCl at room temperature.

### 2.8. Membrane Performance Tests

**Figure 1:**
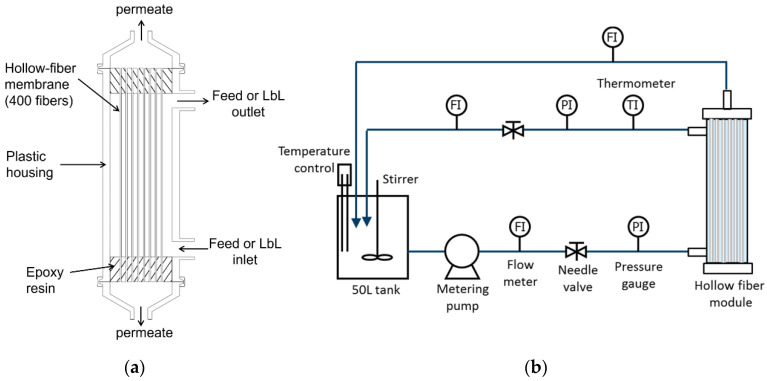
A schematic representation of (**a**) the hollow-fiber membrane module and (**b**) the crossflow evaluation set-up. Layer-by-Layer (LbL) and cross-linking treatments were performed inside the hollow-fiber module.

In this study, we performed a fundamental low-pressure RO test for prepared hollo-fiber membrane at a feed water pressure of 5 bar at 25 °C using the crossflow filtration apparatus shown in Figure 1. Low-pressure RO membranes are important because their use is more energy efficient than high-pressure RO. They are utilized in many applications such as removal of low-molecular weight contaminants from wastewater and toxic heavy metal ions from groundwater sources [26,27,28]. The feed concentrations of salts and neutral solutes were set at 1500 mg/L and 200 mg/L, respectively. Salt concentrations were measured using a conductivity meter (DS-72, Horiba, Kyoto, Japan). Concentrations of neutral solutes were measured by TOC analyzer (ON-LINE TOC-VCSH, Shimadzu, Kyoto, Japan). The rejection of salts and neutral solutes (*R*) and the water permeate flux (*J*_w_) were calculated using Equations (1) and (2), respectively:

(1) $$R=\frac{C_{f}-C_{p}}{C_{f}}\times 100$$

where *C*_f_ and *C*_p_ are the feed and the permeate concentration, respectively.

(2) $$J_{w}=\frac{\Delta V}{A_{m}\cdot \Delta t}$$

where ∆*V* is the permeate volume collected during sampling time ∆*t*, and *A*_m_ is the outer surface area of the hollow-fiber membrane sample. The pure water permeance (*L*_p_) was evaluated by measuring *J*_w_ using distilled water as a feed and calculated by Equation (3):

(3) $$L_{p}=\frac{J_{w}}{\Delta P}$$

The molecular weight cut-off (MWCO; molecular weight at which *R* = 90%) was obtained was estimated by fitting Equation (4) to the rejection data for neutral solutes [29]:

(4) $$R=\frac{1}{{\left(1+M_{w}/B\right)}^{C}}$$

where *M*_w_ is the molecular weight and *B* and *C* are fitting parameters. Alkaline resistance tests for the hollow-fiber membranes were performed by immersing the membrane module in NaOH solutions of pH 10 and 12. The pH values in the module were checked daily with a pH meter and kept constant within ±0.05.

## 3. Results and Discussion

### 3.1. Characterization of Hollow-Fiber Membrane Supports

**Figure 2:**
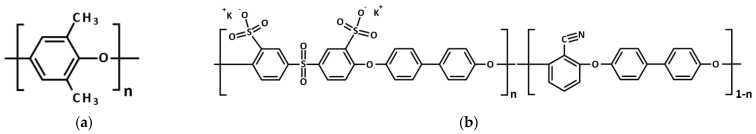
Chemical structures of (**a**) polyphenylene oxide (PPO) and (**b**) sulfonated poly(arylene ether sulfone nitrile) (SPN-20). The hollow-fiber support was made of PPO and the separation layer of SPN-20 was formed on the outer surface of a PPO hollow-fiber membrane. Details are described in Ohkame et al. [22].

**Figure 3:**
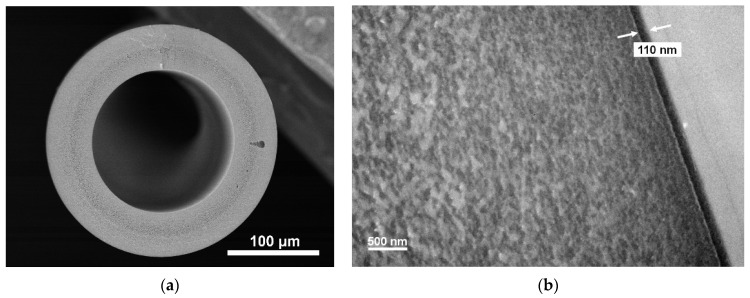
Scanning electron microscopy images of SPN-20/PPO hollow-fiber membranes: (**a**) entire cross-sectional view and (**b**) cross-section near the outer surface of the membrane. The thickness of the SPN-20 layer was 110 nm.

The hollow-fiber membrane used in this study was fabricated at Toyobo Research Center. The nanofiltration (NF) separation layer of the membrane was comprised of sulfonated poly(arylene ether sulfone nitrile) (SPN-20). The hollow-fiber support was made of polyphenylene oxide (PPO). The chemical structures of SPN-20 and PPO are shown in Figure 2. The cross-section images of the SPN-20/PPO hollow fiber are shown in Figure 3. The detailed fabrication method is described elsewhere [22]. The SPN-20/PPO thin-film composite hollow-fiber membrane exhibited excellent NF performance and had a strongly anionic surface through the presence of sulfonate groups, which is preferable for a substrate for polyelectrolyte adsorption. The properties of the SPN-20/PPO membranes are listed in Table 1.

### 3.2. Characterization of CPVA Copolymer

**Figure 4:**
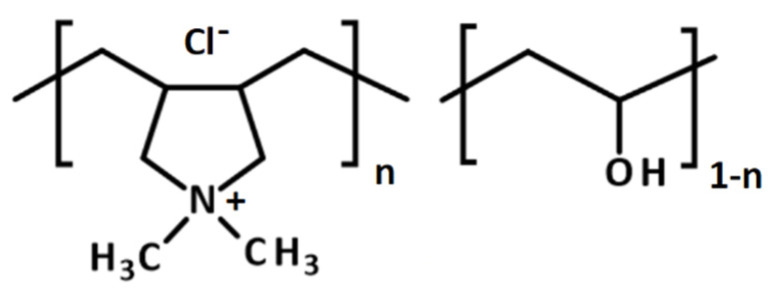
The chemical structure of poly(vinyl alcohol-*co*-diallyldimethylammonium chloride) (CPVA).

**Figure 5:**
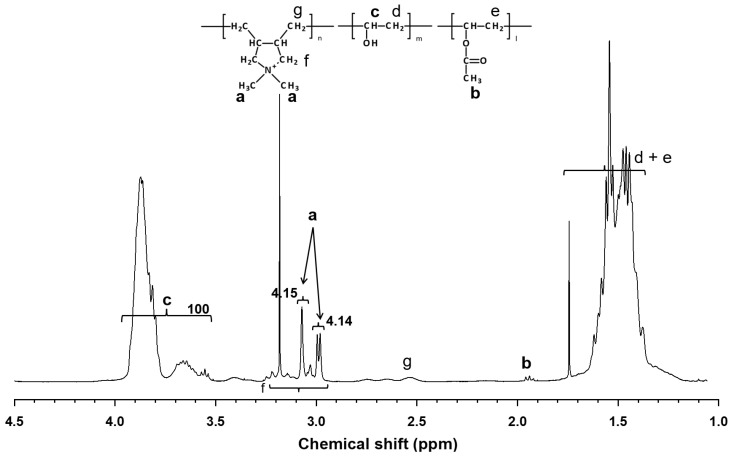
^1^H-NMR spectrum of poly(vinyl alcohol-*co*-diallyldimethylammonium chloride) (CPVA). The degree of cationization (DC) and the degree of saponification (DS) were 1.36 mol% and 99.85%, respectively.

The chemical structure of CPVA synthesized in this study is shown in Figure 4 and its ^1^H-NMR spectrum is shown in Figure 5, where characteristic peaks of PVA are observed at 1.3–1.8 ppm (methylene in VA and VAC), 1.9–2.0 ppm (methyl of acetyl group in VAC), and 3.5–4.0 ppm (methine in VA). The degree of saponification (DS) of the PVA unit in the CPVA copolymer was calculated as:

(5) $$\mathrm{DS}\left(\%\right)=100\times \frac{c}{c+b/3}$$

The peak at 1.9–2.0 ppm (acetyl group in VAC) was faint after saponification and the DS was calculated to be 99.85%. Therefore, the polymer studied here is practically regarded as a two-component random copolymer. The characteristic two peaks of DADMAC unit were observed at 2.98 and 3.07 ppm, which were assigned to protons of two methyl groups in the pyrrolidinium ring [30]. Broad peaks of protons assigned to methine in DADMAC unit were observed at 2.5–2.8 ppm. Other peaks of methylene in DADMAC unit were not observed because of overlapping peaks of PVA. These overlapping peaks were ignored in calculating the copolymer composition for simplicity. Then, the degree of cationization (DC) of the copolymer was calculated as:

(6) $$\mathrm{DC}\left(\%\right)=100\times \frac{a/6}{c+b/3+a/6}$$

The DC of the CPVA was 1.36 mol%. The weight average and the number average molecular weight by GPC were 61,000 and 38,600, respectively.

### 3.3. Adsorption Behavior of CPVA on Anionic SPN-20 Hollow-Fiber Surfaces

**Figure 6:**
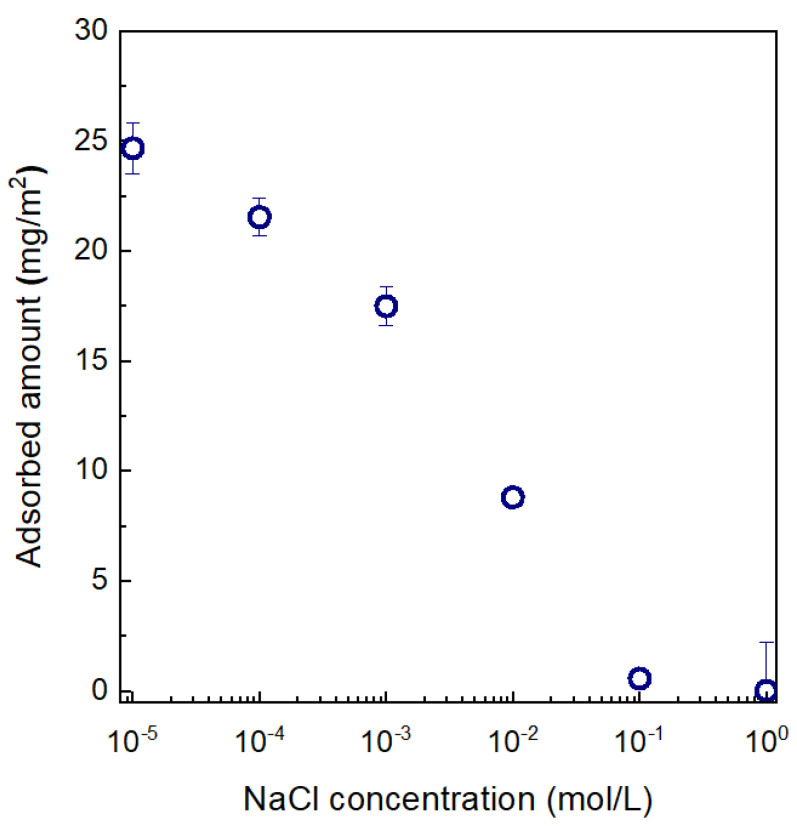
The dependence of the adsorbed amount of CPVA (DC = 1.36 mol%) on NaCl concentration, *C*_s_. The plot at *C*_s_ = 10^−5^ mol/L is the result for no salt addition (the conductivity is less than 1 μS/cm), which was regarded as *C*_s_ = 0 mol/L. Error bars indicate standard deviations.

The effect of ionic strength on the adsorption behavior of CPVA (DC = 1.36 mol%) on the anionic outer surface of SPN-20/PPO hollow-fiber substrate was investigated using NaCl as the added salt. The result is shown in Figure 6. The adsorbed amount of CPVA clearly decreased with increased salt concentration. This behavior is consistent with a screening-reduced adsorption regime as defined by van de Steeg et al. [18]. They conducted an extensive numerical calculation of the adsorption behavior of polyelectrolytes on oppositely charged surfaces using mean-field lattice theory in which the conformation of the adsorbed polymer was modeled by a step-walk in the lattice weighted by the contact energy, the electrical potential, and the mixing entropy. In this regime, the electrostatic attraction between charged polymer segment and the surface is dominant and non-electrostatic interaction is very weak or negligible. The increased salt concentration screens the attractive force between the polymer and surface, which results in decreased adsorption of polymer. Their simulation results also showed that the maximum adsorption of low-charge polyelectrolytes would occur at DC = 1–2% when the ionic strength is low (i.e., *C*_s_ = 10^−5^ mol/L). At *C*_s_ = 10^−5^ mol/L (i.e., pure water), the maximum adsorbed amount of ca. 25 mg/m^2^ was obtained at one-step adsorption, which is equivalent to a thickness of 25 nm if we assume the density of the final XCPVA separation layer is 1 g/cm^3^. This thickness value seems quite promising because the active (top) layer thickness of interfacially polymerized PA separation layer is reported to be 20–40 nm [31,32,33].

### 3.4. Analysis of XCPVA Layer by AFM and TEM

**Figure 7:**
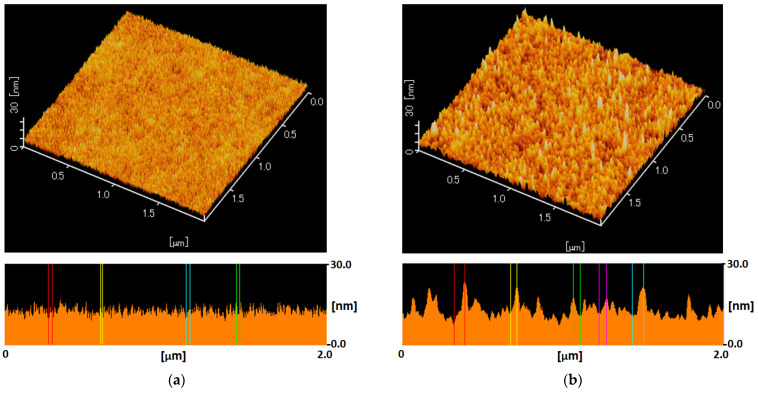
Atomic force microscopy images of the outer surface of the hollow-fiber membrane before and after CPVA adsorption and cross-linking: (**a**) the SPN-20 surface of the hollow-fiber membranes support. The average surface roughness *R*_a_ = 1.26 nm. (**b**) The cross-linked CPVA (XCPVA) surface adsorbed on SPN-20 surface. *R*_a_ = 1.73 nm.

**Figure 8:**
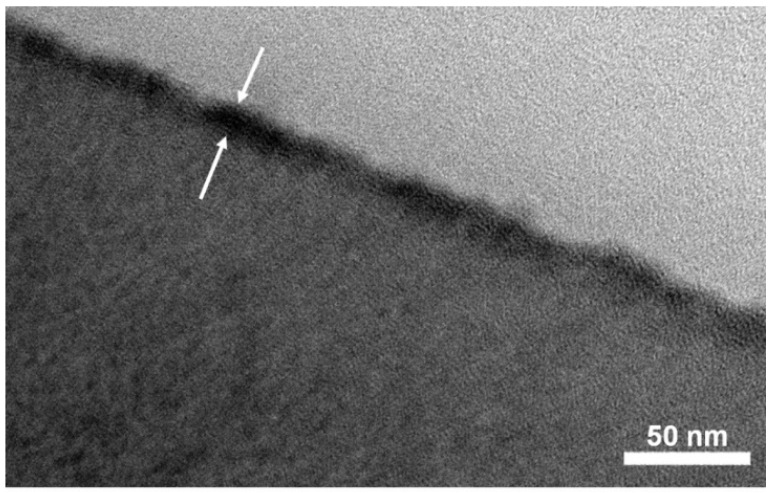
Transmission electron microscopy image of the outermost area of the hollow-fiber membrane after CPVA adsorption and cross-linking. White arrows indicate the XCPVA separation layer stained by titanium element. The thickness of the XCPVA layer was ca. 20 nm.

According to the simple one-step immersion procedure described in the experimental Section 2.4, the CPVA was successfully adsorbed on SPN-20/PPO hollow-fiber membrane surface. Cross-linking with glutaraldehyde is essential to insolubilize and tighten the CPVA separation layer. Figure 7 shows AFM images of hollow-fiber membranes before and after CPVA adsorption and cross-linking. Before CPVA adsorption, the SPN-20 surface was smooth and average roughness *R*_a_ was 1.26 nm. In contrast, the XCPVA surface was slightly rougher than the SPN-20 surface (*R*_a_ = 1.73 nm) and particulate structures were observed that may correspond to a flock of CPVA polymer coils. These *R*_a_ values are much smaller than typical polyamide membranes, which have a characteristic “ridge and valley” structure with *R*_a_ values of 45–50 nm [34,35]. A TEM image of the XCPVA layer stained by titanium is shown in Figure 8. The XCPVA separation layer was very thin, and the thickness was estimated to be ca. 20 nm, which is approximately consistent with the adsorption data in Figure 6 (thickness of 25 nm when the density is 1 g/cm^3^). The XCPVA layer was formed on top of the SPN-20 layer and no clear migration of CPVA into the SPN-20 layer was observed. This was because the MWCO of the SPN20/PPO hollow-fiber support was 890 and its pore size was small enough to sieve CPVA molecules during the adsorption step.

### 3.5. Zeta Potential of PPO, SPN-20, and Cross-Linked CPVA Surfaces

**Figure 9:**
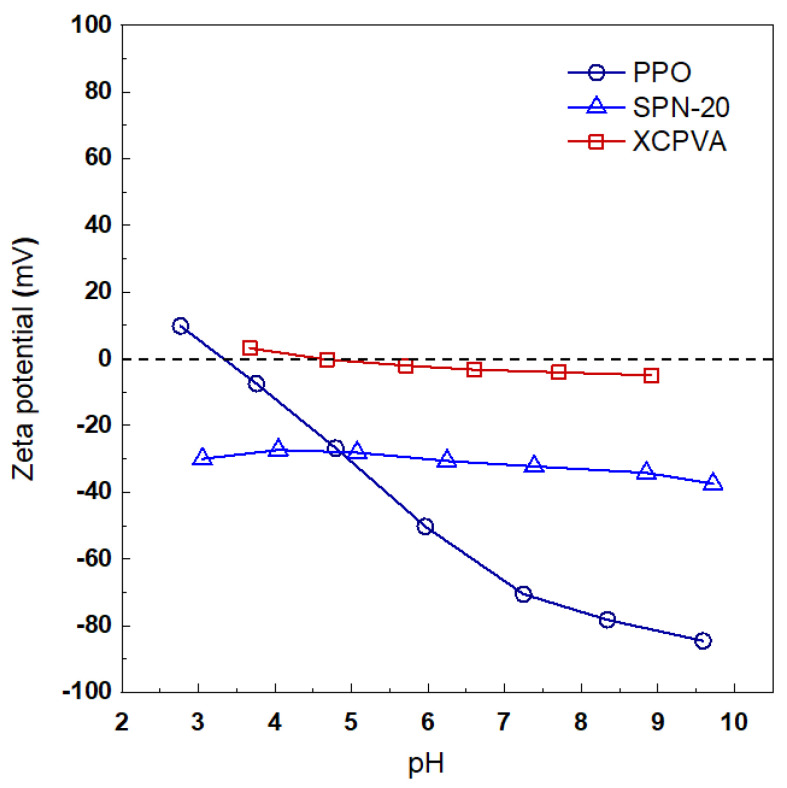
Zeta potential of polymeric films of PPO, SPN-20, and cross-linked CPVA layer formed on SPN-20 (XCPVA) as a function of pH measured in 1 mM KCl solution.

The zeta potentials of membrane materials investigated in this study were evaluated using polymeric films made from PPO, SPN-20, and XCPVA formed on an SPN-20 film. Unfortunately, it was difficult to perform reproducible measurements of outside-in type hollow-fiber membranes because of their complex shape. Figure 9 shows the zeta potentials of these polymeric films. The PPO film showed a characteristic profile for non-ionic hydrophobic surface [36]. At pH 3, PPO showed a slightly positive zeta potential, and its isoelectric point was at pH 3.5. With increasing pH, the surface of PPO film became increasingly negatively charged and reached −85 mV at pH 9.6. This behavior is explained by the preferential adsorption of hydroxide ions on the hydrophobic surface [37]. In contrast, SPN-20 film showed almost constant negatively charged character at around −30 mV irrespective of pH because of the existence of strongly negative sulfonate groups in SPN-20. The XCPVA layer formed on SPN-20 showed neutral character despite having quaternary ammonium cationic groups. This was possibly because of the low cationic content (DC = 1.36 mol%) of the CPVA copolymer. Partially acetalized hydrophilic PVA structure was responsible for the neutral zeta potential profile.

### 3.6. Separation Performance of XCPVA Hollow-Fiber Membrane

**Figure 10:**
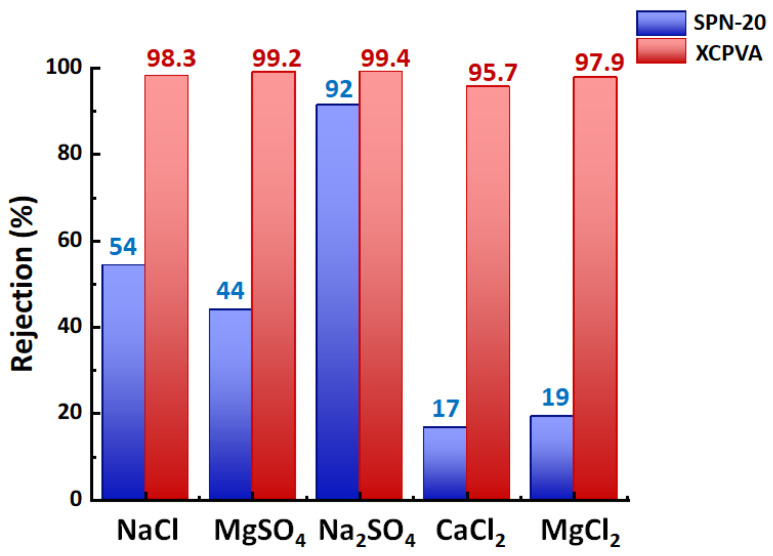
Salt separation performance of hollow-fiber membranes before and after CPVA adsorption and cross-linking (XCPVA). The feed pressure and temperature were 5 bar and 25 °C. Salt concentrations were 1500 mg/L. Pure water permeances of the SPN-20/PPO membrane and XCPVA-modified membrane were 3.9 L∙m^−2^∙h^−1^∙bar^−1^ and 0.24 L∙m^−2^∙h^−1^∙bar^−1^, respectively.

**Figure 11:**
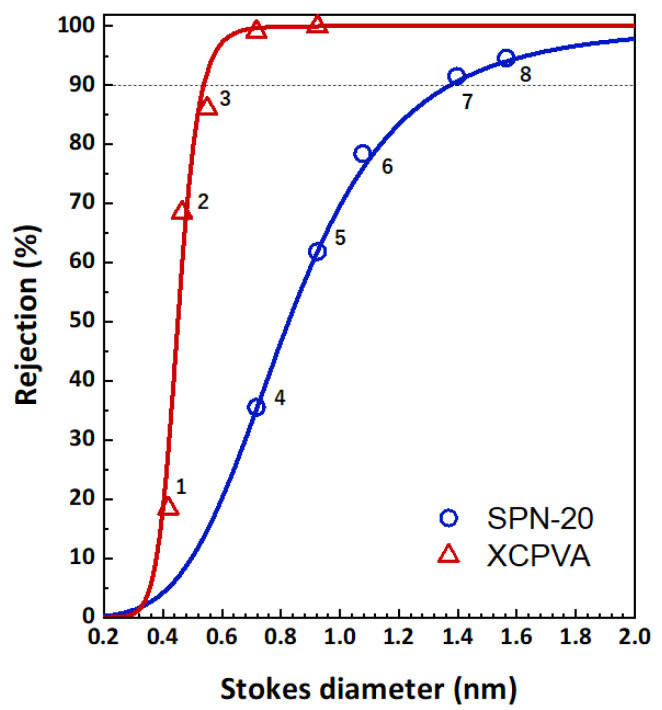
Neutral solute separation performance of hollow-fiber membranes before and after CPVA adsorption and cross-linking (XCPVA). The feed pressure and temperature were 5 bar and 25 °C. Solute concentration was 200 mg/L. Solid lines represent fitting curves according to Equation (4). MWCO values of the SPN-20/PPO hollow-fiber membrane and the XCPVA-modified membrane are 890 and 92, respectively.

The salt rejection performances of SPN-20/PPO hollow-fiber membranes and XCPVA-modified hollow-fiber membranes are shown in Figure 10. The ion rejection effect was drastically improved by the formation of the XCPVA separation layer. Excellent rejection values for 1:1 type salt (NaCl, *R* = 98.3%) and 1:2 type salt (MgSO_4_, *R* = 99.2%; Na_2_SO_4_, *R* = 99.4%) were obtained under low-pressure RO test conditions (5 bar, 1500 mg/L). However, rejection of 2:1 type salts (CaCl_2_, *R* = 95.7%; MgCl_2_, *R* = 98.3%) was less effective than for 1:1 and 1:2 type salts, which may reflect the separation characteristics of the strongly negatively charged SPN-20 support layer because it showed rejection values of only 17% and 19% for CaCl_2_ and MgCl_2_. For comparison, the NaCl rejection of a commercial low-pressure polyamide RO membrane (XLE from DuPont) evaluated under similar low-pressure RO test conditions (5 bar, 2000 mg/L) is reported to be 98.6% [38]. Therefore, the present result of XCPVA membranes is very promising as a novel RO membrane material. Typical NaCl rejections of LbL-type membranes reported in the literature are around 20% to 90% [10,11,39]. Figure 11 shows MWCO curves for the same membranes. The hydrodynamic (Stokes) diameters of markers used for the MWCO evaluation are listed in Table 2. Stokes diameters were calculated using Equation (7) according to the approximation by Bowen et al. [40]:

(7) $$\log_{10}r_{s}=-1.3363\times 0.395\log_{10}M_{w}$$

where *r*_s_ is Stokes radius and *M*_w_ is molecular weight. The SPN-20/PPO hollow-fiber membrane was a typical nanofiltration membane with an MWCO value of 890, which corresponds to a Stokes diameter of 1.37 nm. In contrast, the XCPVA-modified membrane showed an MWCO of 92 and corresponding Stokes diameter of 0.54 nm, which is comparable with commercial polyamide RO or tight NF membranes [41]. As shown by the Zeta potential data (Figure 9), the SPN-20 layer exhibited a negatively charged character and its ion separation performance was highly dependent on electrostatic interaction (i.e., Donnan exclusion), whereas the XCPVA layer showed a neutral Zeta potential despite of the presence of quaternary ammonium moieties in the CPVA and the molecular sieving effect became more dominant than the Donnan exclusion effect. These results indicate that the small amount of cationic groups in the CPVA solely play a role for electrostatic adsorption on the SPN-20 surface, and the RO separation character is derived from cross-linked PVA network structures. It is noteworthy that the XCPVA separation layer formed via the simple LbL treatment is defect-free and physically durable. We observed no deteoriration of XPVA layers over a period of at least 3 months of immersion in water and RO tests, which indicates that XCPVA is a promising RO material.

### 3.7. Alkaline Resistance of XCPVA-Modified Hollow-Fiber Membrane

**Figure 12:**
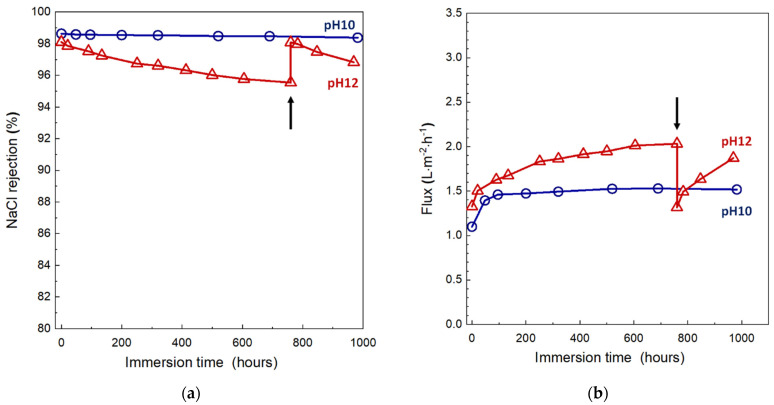
Alkaline resistance of the XCPVA-modified hollow-fiber membranes at pH 10 and 12: (**a**) NaCl rejection, (**b**) flux. The black arrows indicate the point at which LbL treatment was repeated.

**Figure 13:**
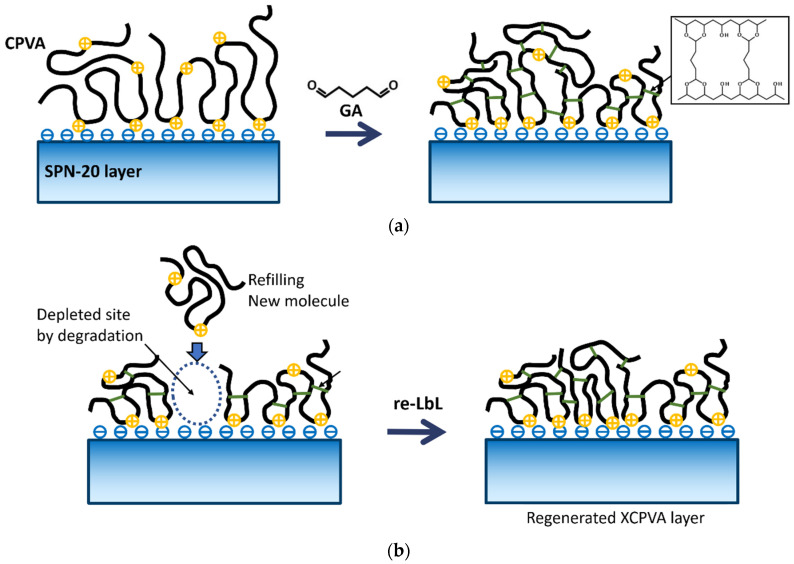
The proposed mechanism of adsorption and cross-linking of low-charge CPVA copolymers on anionic SPN-20 surfaces. (**a**) Initial adsorption of CPVA on SPN-20 followed by glutaraldehyde (GA) cross-linking. The inset shows the cross-linked PVA structure which forms 1,3-dioxane rings bridged by C3 spacers. (**b**) Regeneration by repeated LbL (re-LbL) treatment in which the depleted sites of XCPVA layer are refilled by new CPVA molecules via electrostatic interactions. Subsequent cross-linking restores the XCPVA layer to a state similar to the virgin membrane.

The stability of RO membranes under alkaline conditions is an important property because alkaline or alkaline-enhanced surfactant cleaning protocols are effective against organic fouling and biofoulings [42,43,44]. Figure 12 shows the change in NaCl rejection and membrane flux of the XCPVA-modified hollow-fiber membranes after immersion in alkaline solutions at pH 10 and 12. In alkaline solution at pH 10, the membrane showed no obvious degradation in NaCl rejection over a total exposure time of about 1000 h. At the start of alkaline immersion tests, however, membrane fluxes significantly increased by about 30%. Currently, we have no experimental evidence to explain the cause of this flux increase. This change might be attributed to some physical pore loosening or changes in XCPVA chemical structure and will be a worthwhile topic of investigation in future work. At pH 12, NaCl rejection of the membrane gradually decreased, possibly because of hydrolysis of XCPVA. After 790 h at pH 12, we performed an LbL treatment (denoted as “re-LbL” in Figure 12), which is the same protocol to prepare the virgin XCPVA-modified membrane. Interestingly, the membrane performance was restored to almost the same rejection and flux levels as the virgin membrane. This phenomenon was possibly caused by the mending effect by electrostatic adsorption of CPVA at the depleted site in the XCPVA layer by alkaline hydrolysis. The schematic representation of the XCPVA formation mechanism and the regeneration of degraded XCPVA are shown in Figure 13.

## 4. Conclusions

A novel hollow-fiber RO membrane was successfully fabricated via the adsorption and cross-linking of CPVA copolymers onto anionic SPN-20/PPO hollow-fiber membranes. The maximum adsorbed amount of CPVA was achieved at very low ionic strength conditions (i.e., no salt addition and no pH adjustment) because of innately low intramolecular repulsion forces of low-charge CPVA copolymers. The cross-linking of hydroxyl groups of PVA moieties by glutaraldehyde enabled the formation of a high-density skin layer. The resultant XCPVA-modified hollow-fiber membrane showed excellent NaCl rejection up to 98.5% and an MWCO of 92 under the low-pressure RO test conditions, which confirmed that the thin separation layer (ca. 20 nm thickness) of XCPVA was free of defects. The alkaline tolerance of the membrane was good. It is noteworthy that regeneration of the degraded XCPVA layer was possible by performing the same adsorption and cross-linking procedure on the damaged membrane. The regenerability of the LbL-type RO membrane is a promising feature that may extend RO membrane lifetimes.

## Figures and Tables

**Table 1:** Properties of SPN-20/PPO hollow-fiber membranes [22].

Outer/Inner Diameter (μm)	Pure Water Permeance (L∙m^−2^∙h^−1^∙bar^−1^)	NaCl Rejection ^1^ (%)	MWCO ^2^ (Da)
250/150	3.9	54	890

Detailed fabrication of membrane described by Ohkame et al. [22]. ^1^ NaCl rejection value was measured at 5 bar and 1500 mg/L. ^2^ MWCO was determined by the rejection curve of glucose, sucrose, raffinose, α- and γ-cyclodextrins. The test condition was at 5 bar and 200 mg/L.

**Table 2:** Molecular weight and Stokes diameter of various solutes.

No.	Solute	Molecular Weight	Stokes Diameter [40] (nm)
1	Ethanol	46.1	0.42
2	2-propanol	60.1	0.46
3	Glycerol	92.1	0.55
4	Glucose	180.2	0.72
5	Sucrose	342.3	0.92
6	Raffinose	504.4	1.08
7	α-cyclodextrin	972.9	1.40
8	γ-cyclodextrin	1297.1	1.56
